# A Closer Look at Periocular Necrotizing Fasciitis: A Systematic Review of Literature

**DOI:** 10.3390/diagnostics15091181

**Published:** 2025-05-07

**Authors:** David Oliver-Gutierrez, Elena Ros-Sanchez, Gloria Segura-Duch, Tirso Alonso, Miguel Ángel Arcediano, Alejandra Herranz-Cabarcos, Jessica Matas, Roberto Castro Seco, R. L. P. van der Veen, Anna Boixadera, José García-Arumí, Joan Oliveres

**Affiliations:** 1 Department of Ophthalmology, Hospital Universitari Vall d’Hebron, 08035 Barcelona, Spain; 2 Department of Ophthalmology, Innova Ocular Verte Barcelona, 08006 Barcelona, Spain; 3 Departament de Cirurgia i Ciències Morfològiques, Universitat Autònoma de Barcelona (UAB), Plaça Cívica, 08193 Barcelona, Spain; 4 Department of Ophthalmology, Centro Oftalmológico Barraquer, 08021 Barcelona, Spain; 5 Institut Universitari Barraquer, Universitat Autònoma de Barcelona (UAB), 08021 Barcelona, Spain; 6 Department of Ophthalmology, Consorci Sanitari Integral Moisés Broggi, Sant Joan Despi, 08970 Barcelona, Spain; 7 Department of Ophthalmology, Hospital Clínic Barcelona, 08036 Barcelona, Spain; 8 Department of Ophthalmology, Hospital de la Santa Creu i Sant Pau, 08025 Barcelona, Spain

**Keywords:** necrotizing fasciitis, eye infections, soft tissue infections, cellulitis, orbit, necrosis, *Streptococcus pyogenes*

## Abstract

**Background**: Periocular necrotizing fasciitis (PNF) is a rare but life-threatening emergency that requires immediate recognition, as delayed diagnosis can worsen patient outcomes. To address this critical issue, we conducted the largest and most comprehensive systematic review to date, providing valuable insights into the diagnosis and treatment of PNF to improve clinical practice and patient prognosis. **Methods**: A search on Pubmed, Scopus, Embase, and WOS from January 2013 to August 2024 was performed. Only the cases of NF affecting the periocular region were included with no age limitations. Article selection and data extraction were performed independently by two investigators to avoid bias. Bias on individual studies is low as they represent case reports or case series, and publication bias is partially addressed including all the large case series even if no individual data could be retrieved. **Results**: The cohort included a total of 183 patients with PNF, with detailed patient-specific data for 107 individuals and only aggregated data for another 76. The average age at diagnosis was 54.2 years, and females constituted 44% of the population sample. Notably, 49.6% of the patients were immunocompromised. *Streptococcus pyogenes* was the predominant causative organism, identified in 79.8% of the cases. Most infections were unilateral (72.1%) without extension beyond the periocular area (54.7%). Most patients (89.6%) underwent surgical debridement alongside intravenous antibiotics. Septic shock occurred in 26.8% of the patients, and the overall mortality rate was 4.9%. Visual acuity was unaffected in 67.5% of the patients, though 18.2% progressed to blindness on the affected side. Reconstructive efforts predominantly involved skin grafting, both free and local pedunculate flaps as well as secondary healing in some instances. **Conclusions**: This systematic review summarizes the understanding of periocular necrotizing fasciitis’ (PNF) demographic trends, clinical manifestations, causative pathogens, and patient outcomes. Vigilance for PNF should be heightened when the clinical assessment of the patient’s eyelids reveals rapidly spreading edema and induration, subcutaneous emphysema, or necrotic bullae and/or eschar. Prompt identification and expedited intervention, including debridement and targeted antibiotic therapy, critically influence prognosis. Despite optimal management, patients may still suffer from significant aesthetic impairment, severe complications such as vision loss, or death due to septic shock.

## 1. Introduction

Necrotizing soft tissue infections affect the fascia and subcutaneous tissue, progressing rapidly and leading to secondary skin necrosis [1,2]. Periocular necrotizing fasciitis (PNF) is exceedingly rare, possibly due to the highly vascularized nature of this region [2]. Its consequences range from facial disfigurement of lesser or greater extent, to even death, with vision loss also being rather common and affecting almost a third of patients [3].

PNF exhibits a slight male predominance, with half of the cases occurring in previously healthy patients [3,4], in contrast to other severe infections like mucormicosis, which often affects diabetic or immunosuppressed patients as opportunistic infections. PNF can also have traumatic, post-surgical, or sinusitis-related etiologies, but almost a third of cases are idiopathic [2,3]. Its onset is often nonspecific, characterized by painful erythema and edema, rapidly progressing fever, and pronounced inflammatory signs, ultimately evolving into blisters and necrosis within a few days. Computed tomography (CT) may reveal early-stage subcutaneous air and inflammation with accompanied subcutaneous and other fat plane necrosis, typically sparing muscle masses [3].

Necrotizing fasciitis (NF) has two distinct types of presentation [2]: the lesser common Type 1, a polymicrobial infection caused by aerobic and anaerobic bacteria, and the more common (80%) Type 2, typically attributed to a single microorganism, with Group B beta-hemolytic streptococcus (*S. pyogenes*) being the most common causative agent in half of the cases [2,3].

The most crucial prognostic factor is early recognition as progression is often very rapid [5], requiring urgent debridement alongside broad-spectrum intravenous antibiotic therapy [3], which often includes beta-lactam antibiotics and clindamycin [2,5]. The mortality rates of PNF range from 8.5% [2] to 14.4% [3] in various series, somewhat lower than the mortality rates of NF in other body regions situated between 12.1 and 45.6% [4,6,7]. A multidisciplinary approach is crucial to prevent or treat septic shock, the leading cause of mortality in these patients [2].

Given the significance of this life-threatening disease, where early identification is crucial, we conducted the largest systematic review to date to enhance understanding and improve clinical outcomes. This review aims to provide a comprehensive understanding of the condition while updating key aspects such as demographic data, clinical presentation, treatment options, and patient outcomes.

## 2. Materials and Methods

We undertook a comprehensive systematic review to identify studies detailing patients with PNF, published in either English or Spanish from 1 January 2013 to August 2024. Our search strategy encompassed a combination of the following keywords and Boolean operators, executed across several databases, namely Scopus, Web of Science, Embase, and PubMed: [“Necrotizing Fasciitis”] AND [(“Ophthalmology”) OR (“Periorbital”) OR (“Periocular”) OR (“Eyelid”)]. Additionally, we cross-referenced and manually reviewed the citations of the primary selected articles.

Studies that specifically focused on or discussed necrotizing infections in the periocular or periorbital region and were accessible in full-text format were included. All the articles discussing patient-specific data were included in our review. However, three articles provided only pooled or aggregated data, and efforts to retrieve individualized data by contacting the corresponding authors did not lead to the retrieval of individual data. Therefore, the articles with aggregated data could only be considered for certain types of analysis, while those offering individual patient data were used for all the analyses in this review.

Our review concentrates on extracting demographic details, associated risk factors, microbiologic results, medical and surgical treatment, occurrence of septic shock, visual outcome, and overall patient outcome. No reported data were omitted. If septic shock and death were not explicitly reported, they were assumed not to have occurred.

The article selection and data extraction were performed independently by two investigators and discussed afterward. This systematic review followed the PRISMA guidelines, and the protocol was registered with the PROSPERO database (ID: CRD42023458417) prior to data collection.

Statistical analyses were conducted using the Stata 17 software. Comparative analyses were performed using the *t*-test, chi-squared test, or Fisher’s exact test, as appropriate.

## 3. Results

### 3.1. Studies Selection

**Figure 1:**
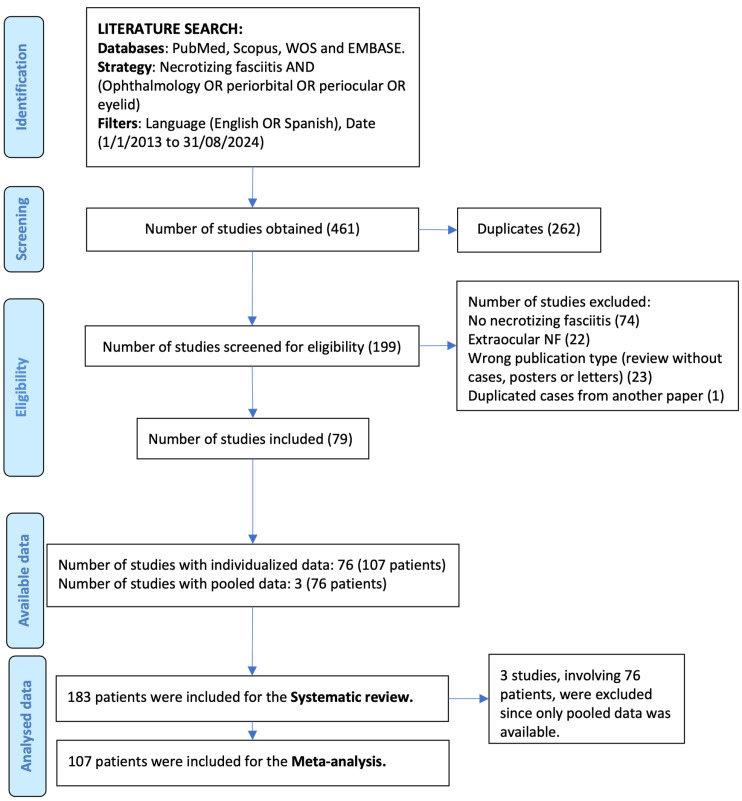
Selection process flow chart.

The literature search yielded 461 studies. Figure 1 depicts the selection process flowchart, leading to 79 studies including a total of 107 patients with individual data and 3 studies that contained pooled data of 76 patients.

All the studies included are summarized in Table 1.

### 3.2. Patient and Necrotizing Fasciitis Features (Table 2)

This systematic review of PNF provided individualized data for 107 cases and pooled data for 183 cases, as summarized in Table 1.

**Causes:** Infections resulting from wounds made up the primary cause with 44.8% of the total. Notably, two distinct cases were linked to medical procedures: one following strabismus surgery [22] and another after the administration of retrobulbar anesthesia [19]. Spontaneous infections occurred in 40.8% of the cases. Skin infections comprised 9.2% of the cases and had varied origins such as hordeolum, pimple picking, varicella zoster infection, and insect bites. Primary sinusitis was responsible for 5.2% of the cases.

**Comorbidities:** Immunosuppression was a standout feature, detected in 49.6% of the studied cases. Specific conditions or treatments leading to immunosuppression included the following: diabetes mellitus (DM) (17.6%), alcoholism or liver cirrhosis (21.5%), and various neoplasms including small cell lung carcinoma, breast adenocarcinoma, endometrial adenocarcinoma, prostate adenocarcinoma, lymphoma, myelodysplasia, and chronic myeloid leukemia. Treatment with corticosteroids and autoimmune diseases like Systemic Lupus Erythematosus, Sjögren syndrome, Ulcerative Colitis, and rheumatoid arthritis were also noted.

**Pathogens:** *S. pyogenes* (Group A Beta Haemolytic streptococcus) was the most prevalent causative pathogen, affecting 79.8% of the cases. *S. aureus* was the second most frequent pathogen present in 15.2% of the cases. *Pseudomonas aeruginosa* was isolated in 4.4% of the cases, and *Klebsiella* sp. accounted for 1.9%. Additional isolated pathogens are detailed in Table 2.

**PNF Extension:** A significant majority, 72.1% of the infections manifested unilaterally, and for 54.7% of patients, the infection remained confined to the periocular region. However, 45.3% of the cases exhibited further facial involvement. Data on whether there was an orbital extension of the NF showed 54.0% of the cases remained preseptal.

### 3.3. Treatment (Table 3)

**Systemic antibiotic:** All the patients received systemic antibiotics, and the most commonly used were penicillins and clindamycin.

**Surgical Treatment:** As seen in Table 3, 89.6% of the patients underwent at least one surgical debridement with around one-third (30.6%) requiring several interventions. A total of 8.7% of the patients presented with more extended disease that invaded the orbit or caused severe ocular damage, resulting in enucleation for 5 patients [13,56,64,68,70] or exenteration on 11 patients [14,58,78,79,82,83].

**Other Medical Therapies:** Negative pressure wound therapy (NPWT) was applied in six cases [4,18,37,45,66] and Hyperbaric oxygen therapy (HBOT) was administered in five patients [30,58,65]. Immunoglobulin G therapy was used in four cases [21,29,33,39].

### 3.4. Outcome (Table 3)

**Vision:** In most patients (67.5%) visual acuity was unaffected. In 14.3% of the cases, visual acuity was affected, and 18.2% of the cases resulted in blindness of the eye on the affected side.

**Septic Shock:** Septic shock was reported in less than one-third (26.8%) of the patients.

**Mortality:** Death occurred in only 4 out of 107 (3.8%) patients from the individualized data group and in 9 out of 183 (4.9%) patients from the pooled data group.

### 3.5. Comparative Analysis

A comparative analysis revealed several noteworthy findings regarding risk factors for serial debridement, blindness, septic shock, and mortality (Table 4). People with advanced age seem to undergo less frequent debridements (*t*-test *p* = 0.083) but had a higher risk of septic shock (*p* = 0.009) and possibly mortality (*p* = 0.058). Gender did not exhibit associations with an increased need for debridement, blindness, or mortality; however, the septic shock group included a notably higher percentage of females. All the deceased individuals were immunosuppressed, in contrast to only 47% of the immunosuppressed patients in the survival group (*p* = 0.05). Similarly, DM was more prevalent in the mortality group (*p* = 0.015). Finally, the patients with extraocular affection could need more debridements (*p* = 0.13) and have higher mortality (*p* = 0.027).

## 4. Discussion

PNF is a rare occurrence but stands out as an alarming medical emergency that mandates prompt attention from ophthalmologists and other healthcare providers. This study was conducted with the primary objective of providing a detailed understanding of PNF, encompassing various aspects such as its epidemiology, clinical presentation, causative pathogens, management strategies, and patient outcomes.

### 4.1. Incidence

Although the overall incidence of PNF is low, some authors suggest an increase in incidence in recent years [76,79]. Among the 107 patients with individualized data, 24 cases were published between 2023 and 2024. Factors contributing to this rise may include an aging population, increased antibiotic resistance, lingering effects of COVID-19 on immune function, and possibly, more frequent reporting in the scientific literature.

### 4.2. Pathophysiology

PNF is marked by an aggressive clinical course [5] triggered by bacterial toxins leading to microvascular thrombosis and rapid local immune response [74]. Clinically significant eyelid edema, disproportionate pain, erythema, and fever are indicative of the extensive local inflammatory response, with systemic implications including septic shock [74]. The periocular region’s unique anatomy contributes to symptom severity including visual impairment but allows a rapid immune response and antibiotic penetration, often resulting in a better prognosis than NF at other sites [74,84].

### 4.3. Diagnosis

Diagnosing PNF is challenging, especially in the early stages. The mean time to diagnosis in this review was 3.7 days (SD of 3.9); however, rapid deterioration can occur in a few hours [5]. Main differential diagnoses include preseptal and postseptal cellulitis [3,36,38,61,62,67], and occasionally angioedema due to its rapid course [8,31,37,47,74].

Other conditions to consider are blepharitis, conjunctivitis [13,16], herpes zoster, erysipelas, granulomatosis with polyangiitis, endogenous endophthalmitis, retrobulbar hemorrhage [19], cavernous sinus thrombosis, and rhino-orbital mucormycosis [3,74].

Early signs of PNF include rapid cellulitis spread, poor antibiotic response, severe local pain, or anesthesia from nerve damage caused by the spreading infection. Other clinical symptoms are subcutaneous emphysema, serosanguineous bullae, or skin necrosis, with patients often developing high fever and low blood pressure [3,10] with lab tests often showing systemic infection indicators [10,30].

Radiological imaging can aid in diagnosis; however, it should not delay the initiation of antibiotics or prompt debridement.

### 4.4. Risk Factors

Roughly half of the cases presented occurred in immunocompromised individuals, consistent with prior reviews [2,3]. Immunosuppression was also significantly associated with increased mortality (*p* = 0.05), and all the patients who died were immunosuppressed in contrast to 47% of those who survived. DM and alcohol use/cirrhosis were present in 17.6% and 21.5% of the patients, respectively, showing a significant association with PNF development compared to the general population 10.5% [85] and 11.8% [86] (*p* = 0.019 and *p* = 0.0022). These diseases could be contributing factors to the development of PNF.

Other risk factors widely recognized include arteriosclerosis, atherosclerosis, HIV infection, corticosteroid therapy, chronic renal failure, cancer, substance abuse, obesity, malnutrition, senescence, and recipients of organ transplants [84]. Other comorbidities are described in Table 2.

### 4.5. Etiology

The primary etiological factor for PNF accounting for almost half of the cases is injury or trauma in the periocular region, including two post-surgical cases [19,22]. The current literature mentions similar percentages [2,3]. The second largest group seems idiopathic in nature, accounting for around 40%, which may involve minor or overlooked injuries as proposed by Amrith et al. [2].

Sinusitis contributed to approximately 5% of the PNF cases, often presenting diagnostic challenges due to the atypical presentation for ophthalmologists due to the absence of dermal injury. In these cases, CT is essential [79]. A total of 65% of the cases in this review utilized CT scans. Notably, imaging should not delay surgical intervention [5].

NF has two distinct types of presentation [2]. The predominance of GABHS in PNF cases (80%) typically characterizes Type 2 NF, known for its lower mortality compared to the polymicrobial Type 1 variant [2,84]. Corroborating this, Yan et al.’s [84] review on head and neck NF highlighted that 76% of the periocular instances were monomicrobial and attributed to GABHS, which may very well explain the lower mortality rates in PNF which we found to be as low as 4.9%, a rate much lower than that reported in general NF situated between 12.1 and 45.6% [4,6,7].

### 4.6. Treatment

Optimal NF management involves the immediate administration of intravenous beta-lactam antibiotics, such as penicillin or cephalosporin, which effectively target GABHS. Clindamycin is recommended alongside these antibiotics for its ability to inhibit protein synthesis and reduce toxin production [2,5]. Antibiotic therapy should be tailored to culture sensitivity results as soon as they become available.

In addition to intravenous antibiotics, surgical debridement is essential for NF treatment as it substantially reduces bacterial load and improves antimicrobial penetration [3,74]. Debridement should be extensive, reaching healthy bleeding tissue, and can be followed by irrigation with saline, hydrogen peroxide, or iodine [5]. When feasible, preserving and repositioning skin flaps after removing necrotic tissue allows for better aesthetic outcomes, as well as preserving the eyelid margin and eyelashes. We found that surgical debridement was performed in nearly 90% of the cases, with one-third requiring multiple procedures, and around 10% necessitating enucleation or orbital exenteration. During or prior to debridement, cultures of the purulent material should be obtained, considering that cultures from the necrotic crust may yield negative results.

While there are instances described in the current literature where patients have recovered favorably without surgical intervention [12,26,54], this strategy is generally reserved for those with very localized and limited PNF, rapidly ceasing growth of erythema or necrotic tissue with antibiotic treatment, and no signs of orbital involvement [3,54,87]. However, we advocate for surgical debridement in all cases, especially when the infection spreads or fails to improve as expected [54]. If surgical debridement is deferred, it is a prudent practice to delineate the affected margins with a visible marker and to monitor closely for any progression [5,79]. Mutamba et al. [16] describe three cases where an initial response to antibiotics delayed the progression of NF, allowing for delayed debridement with possibly more favorable reconstructive outcomes. This was mirrored in one of our previously published cases [79], where debridement was delayed for two weeks due to the large extension and a positive response to antibiotics.

It is worth highlighting that the group with fewer debridements had a significantly higher average age. Older age was also more frequent in the shock and mortality group. One plausible hypothesis for this correlation could be that older patients undergo fewer debridements, potentially contributing to higher mortality rates. However, it is essential to acknowledge that there are multiple factors contributing to an increased risk of shock and mortality in older patients, and while this finding is intriguing, it requires further investigation.

The efficacy of hyperbaric oxygen therapy (HBOT) and negative pressure wound therapy (NPWT) in PNF treatment remains debated [2,30]. HBOT, used in five cases in this review [30,58,65], may reduce ischemic damage and aid healing by increasing oxygen levels in the affected tissues, with possible bactericidal effects [30,88]. NPWT, applied in six cases [4,18,37,45,66], is believed to support healing by reducing swelling and bacterial load, improving drainage, and encouraging tissue regeneration [37]. Studies indicate NPWT is safe in the periocular area and may expedite recovery while reducing repeat debridements [18]. Importantly, these therapies should not delay necessary debridement and are best used as adjuncts to antibiotics and surgery. Contreras et al. applied −125 mmHg in adults and −75 mmHg in infants, with an average treatment duration of 6.7 days [18]. Similarly, Gillespie et al. reported the use of −75 mmHg continuous suction in a periocular case [37], with ocular dressings changed every 2–3 days [18,37].

On the other hand, intravenous immunoglobulins, with immunomodulatory and anti-inflammatory properties, have been used in four PNF cases [21,29,33,39]. These agents facilitate the antibody-mediated neutralization of bacterial superantigens and toxins.

### 4.7. Outcome

The primary sequelae of PNF are minor aesthetic defects and eyelid malfunctions, including lagophthalmos or ectropion post-surgical debridement and reconstruction, managed with skin grafts or local flaps like glabellar, Tripier, or temporal flaps. In some instances, secondary intention healing may have good results [80]. Blindness in the affected eye, occurring in 18% of the cases, is a severe but less common outcome, caused by endophthalmitis [26,61], central retinal artery occlusion [31,72], or evisceration/enucleation.

Furthermore, PNF mortality rates appear to be declining from 14.4 to 4.9% from the year 1950 up to the present (Table 5) [2,3], being lower than other body parts [4,6,7]. This fortunate trend coincides with advancements in the available antibiotic treatment and probably increased awareness leading to the earlier recognition of PNF, with higher mortality rates observed in older adults and females (Table 4).

### 4.8. Other Reviews (Table 5)

In their six-decade retrospective review published in 2009, Lazzeri et al. [3] analyzed 103 PNF cases with a median age of 50.2 years (range 17 months to 93 years). Similarly, Amrith et al. [2] conducted a two-decade review published in 2013 of 94 patients, with a median age of 46.3 years (range 0.1 to 83 years). Both median ages were slightly lower than our study’s median age of 54.2 years (Table 1). Gender distribution was comparable across all the studies, with group A beta-hemolytic Streptococcus (GABHS) identified as the primary pathogen in 51% to 80% of the cases. Notably, our review observed slightly higher blindness rates, a lower incidence of septic shock, and a significantly lower mortality rate of 4.9%.

Other authors have attempted systematic reviews [78,89]; however, the limited number of cases identified suggests an insufficient literature search, highlighting the need for a more thorough and comprehensive review.

### 4.9. Strengths and Limitations

For this review, we conducted a comprehensive search across four databases to capture all instances of PNF reported in the literature. Our search strategy aimed to include all the relevant studies, but it is possible that some studies not indexed in these databases or published in non-indexed journals may have been missed. To minimize bias, the article selection and data extraction were performed independently by two investigators. Despite these efforts, the potential for human error remains, particularly in large databases where information extraction and typographical errors can occur.

Other common limitations of systematic reviews are the heterogenicity of studies, lack of access to complete data, and publication bias. To address these, we included both individual cases and aggregated data from case series, ensuring a robust sample. While aggregate data may be limited in comparative analysis (as in Table 4), it holds descriptive value. We acknowledge that publication bias might skew results toward more severe or notable cases, but incorporating large case series helped mitigate this bias by including cases less likely to be published individually.

## 5. Conclusions

PNF is a very rare entity. Its suspicion should be high in cases exhibiting edema and induration beyond the erythematous zone, crepitus, or air in imaging and necrotic blisters or eschar. Early recognition, followed by immediate and appropriate intravenous antibiotic therapy, followed by prompt surgical debridement, is the most important positive prognostic factor as progression can be very rapid. Aesthetic sequelae are common. Other severe complications, such as vision loss or septic shock, occur more rarely but can be life-threatening.

## Figures and Tables

**Table 1:** Characteristics of study participants included in the systematic review and meta-analysis by studies.

Authors (Year)	*N*	Age (Range)	Sex	Vision Loss *	Septic Shock	Death
Franzen et al. (2013) [8]	1	31	M	Not affected	No	No
Saonanon et al. (2013) [9]	1	48	M	Not affected	No	No
Shah et al. (2013) [10]	1	22	M	Not affected	No	No
Richir et al. (2013) [11]	1	43	F	Not affected	Yes	No
Mehta et al. (2013) [12]	1	57	F	Not affected	No	No
Rodríguez-González et al. (2013) [13]	1	53	M	Blindness	No	No
Shield et al. (2013) [14]	5	55.6 (42–62)	3F, 2M	1 Not affected 4 Blindness	2 Septic Shocks	No
Arazi et al. (2023) [15]	1	66	F	Affected	Yes	No
Mutamba et al. (2013) [16]	3	67 (57–76)	1F, 2M	All not affected	No	No
Casey et al. (2014) [17]	1	46	M	Not affected	Yes	No
Contreras-Ruiz (2014) [18]	2	53 (48–58)	M	Not affected	No	No
Gelaw et al. (2014) [19]	1	33	F	-	No	-
Günel et al. (2014) [20]	1	75	M	Not affected	No	No
Brissette et al. (2014) [21]	1	34	M	Not affected	No	No
Yau et al. (2015) [22]	1	2	M	Not affected	No	No
Khurana et al. (2015) [23]	6	8 m (5–11 m)	2F, 4M	-	No	No
Jain et al. (2015) [24]	1	6	M	-	Yes	No
Abdul Kadir et al. (2016) [25]	1	72	M	-	Yes	Yes
Hagiya et al. (2016) [26]	1	62	F	Blindness	Yes	No
Danan et al. (2016) [27]	1	50	M	-	No	No
Wolkow et al. (2017) [28]	1	64	M	Not affected	No	No
Uhrich et al. (2017) [29]	1	64	F	-	Yes	Yes
Singam N et al. (2017) [30]	1	60	F	Not affected	No	No
Sultan et al. (2017) [31]	1	50	M	Blindness	No	No
Zhang et al. (2017) [32]	1	56	M	Not affected	No	No
Eiben & Rodriguez-Villar (2018) [33]	1	60	M	Not affected	Yes	No
Leach et al. (2018) [34]	1	70	M	Not affected	No	No
Leonardo et al. (2018) [35]	1	55	F	Not affected	No	No
Jaffer et al. (2018) [36]	1	51	F	Not affected	Yes	No
Gillespie et al. (2018) [37]	1	44	M	Not affected	No	No
Proia (2018) [38]	1	1	M	-	No	No
Herdiana et al. (2018) [39]	1	69	F	Not affected	Yes	No
Olsson et al. (2018) [40]	1	12	M	Not affected	No	No
Deneubourg et al. (2018) [41]	1	30	F	Not affected	Yes	No
Setiawati et al. (2018) [42]	1	4	F	Blindness	Yes	No
Park et al. (2019) [43]	1	53	F	-	1	No
Bermudez & Walsh (2019) [44]	1	58	F	-	No	No
Karan et al. (2019) [1]	1	81	F	-	Yes	Yes
Cozzupoli (2019) [45]	1	70	F	-	Yes	Yes
Tong et al. (2019) [46]	1	51	M	Not affected	No	No
Placinta et al. (2019) [47]	1	80	F	Not affected	Yes	No
Cereceda-Monteoliva et al. (2019) [48]	1	56	M	-	Yes	No
Nadal et al. (2019) [49]	1	32	F	Not affected	No	No
Sud et al. (2019) [50]	1	52	F	-	No	No
Mehraban et al. (2019) [51]	1	51	F	-	Yes	No
Landeen et al. (2020) [52]	1	58	F	Affected	No	No
McCabe et al. (2020) [53]	1	56	F	Not affected	Yes	No
Kontou et al. (2020) [54]	1	42	M	Not affected	No	No
Negi et al. (2020) [55]	1	32	M	Affected	No	No
Muthie et al. (2020) [56]	1	42	F	Not affected	No	No
Compton et al. (2020) [57]	1	44	F	Not affected	Yes	No
Würtz et al. (2020) [58]	6	55.8 (37–85)	4F, 2M	1 Not affected 3 Blindness 2 -	2 Septic Shocks	No
Ting et al. (2020) [59]	1	35	F	Not affected	No	No
Pereira et al. (2021) [60]	1	66	F	Not affected	Yes	No
Lee et al. (2021) [61]	1	62	M	Blindness	No	No
Cameron et al. (2021) [62]	1	25	F	Affected	Yes	No
Zhou et al. (2021) [63]	1	59	M	Not affected	No	No
Haque et al. (2021) [64]	2	65 (62–68)	1F, 1M	Blindness -	No	No
Yazici et al. (2021) [65]	1	70	M	Blindness	No	No
Reddy et al. (2021) [66]	1	44	M	Not affected	Yes	No
Rossetto et al. (2021) [67]	1	68	M	Not affected	No	No
Tartar et al. (2022) [68]	1	33	M	Not affected	No	No
Silverman et al. (2022) [69]	1	21	M	Not affected	No	No
Kakimoto et al. (2022) [70]	1	55	F	-	No	No
Suh et al. (2022) [71]	1	43	M	Not affected	No	No
Mosenia et al. (2022) [72]	1	39	M	Blindness	No	No
Gaur et al. (2023) [73]	1	35	M	Not affected	Yes	No
Hadizamani et al. (2023) [74]	1	69	M	-	Yes	No
Ang et al. (2023) [75]	1	33	F	Not affected	No	No
Schuh et al. (2023) [76]	5	71 (65 to 83)	5M	4 Not affected 1 Blindness	4 Septic Shocks	No
Huang et al. (2023) [77]	1	26	M	Not affected	No	No
Pertea et al. (2023) [78]	1	67	M	Blindness	Yes	No
Oliver-Gutierrez et al. (2024) [79]	9	67 (41 to 82)	5F, 4M	6 Not affected 2 Affected 1 Blindness	2 Septic Shocks	No
Arun et al. (2024) [5]	2	66 (52 to 69)	2M	2 Affected	1 Septic Shocks	No
Blanchard et al. (2024) [80]	1	75	M	Not affected	No	No
Hojjatie et al. (2024) [81]	1	79	F	Affected	No	No
**Totals only individualized data:**	107	Median 55.5 (8 m–85 y) Mean 50.9 (SD 22.3)	46F, 61M	56 (67.5%) Not affected 8 (9.6%) Affected 19 (22.9%) Blindness	38 Septic Shocks (35.5%)	4 (3.8%)
Wladis et al. (2015) [82]	17	48.1	8F, 9M	9 Not affected 5 Affected 3 Blindness	No	1 Death
Flavahan et al. (2014) [83]	30	68	17F, 13M	15 Not affected 9 Affected 2 Blindness 4 -	5 Septic Shocks	3 Death
Rajak et al. (2016) [4]	29	56	9F, 20M	24 Not affected 4 Blindness 1 -	6 Septic Shocks	1 Death
**Totals with pooled data:**	183	Mean 54.2	80F, 103M	104 (67.5%) Not affected 22 (14.3%) Affected 28 (18.2%) Blindness	49 Septic Shocks (26.8%)	9 (4.9%)

* vision loss was classified as follows: not affected when no vision loss was reported, affected when vision loss was reported but higher than Hand Movement, and blind for Hand Movement or less as well as enucleation or exenteration.

**Table 2:** Characteristics of study participants and PNF features.

	Individualized Data (107)		Pooled Data (183)	
	*n*	%	*n*	%
**Cause**	99		174	
Spontaneous	42	42.4%	72	40.8%
Wound or surgery ^a^	38	38.4%	78	44.8%
Sinusitis	9	9.1%	9	5.2%
Other skin infections ^b^	10	10.1%	16	9.2%
**Comorbidities**	107		183	
DM	19	17.6%	-	
AHT (Arterial hypertension)	16	15.0%	-	
Alcoholism or cirrhosis	23	21.5%	-	
Inmonusppressed ^c^	52	48.6%	90	49.6%
No comorbidities ^d^	38	35.9%	-	
**Pathogen ^e^**	100		158	
*S. pyogenes*	74	74.0%	126	79.8%
*S. aureus*	18	18.0%	24	15.2%
*Pseudomona aureginosa*	4	4.0%	7	4.4%
*Klebsiella pneumoniae*	3	3.0%	3	1.9%
Other	13	13.0%	14	8.9%
**Laterality**	99		129	
Unilateral	73	73.7%	93	72.1%
Bilateral	26	26.3%	36	27.9%
**Extraocular**	100		159	
Limited to periocular region	59	59.0%	87	54.7%
Facial involvement	41	41.0%	73	45.3%
**Orbital extension**	87		100	
Preseptal	45	51.7%	54	54.0%
Postseptal	42	48.3%	46	46.0%

(a) Two cases were linked to medical procedures: one following strabismus surgery and another after receiving retrobulbar anesthesia. (b) Other skin infections were attributed to various causes such as hordeolum, pimple picking, varicella zoster infection, or insect bites. (c) Immunosuppression included the following conditions or treatments: diabetes mellitus (DM), alcoholism, cirrhosis, cancer, treatment with corticosteroids, and autoimmune diseases (Systemic Lupus Erythematosus, Sjogren’s syndrome, fibromyalgia, and rheumatoid arthritis). (d) Other comorbidities beyond the table’s scope include Chronic Obstructive Pulmonary Disease (COPD), cardiac conditions (ischemic heart disease, congestive heart failure, and treated pulmonary valve stenosis), hypothyroidism, hematological disorders (thrombocytopenia, end-stage renal failure, and Waldenström’s macroglobulinemia), syphilis, various cancers (breast, lung with metastases, endometrial, and liver), and lifestyle-related factors (obesity, smoking, substance abuse, malnutrition, and homelessness). (e) In the bacterial findings, twelve instances of *S. pyogenes* were found alongside *S. aureus*; of these, one was paired with MRSA and one was also found with *S. epidermidids*. Another case was found alongside Propionibacterium Acnes, and yet another with Streptococcus Constellatus. Additionally, out of six *S. aureus* cases, three were identified as MRSA. One of these *S. aureus* cases coexisted with Candida Albicans; another was found with both *S. parasanguinis* and Enterobacter Cloacae. The other bacteria and pathogens identified included *Anthrax*, *Aspergillus*, *Burkholderia pseudomallei*, polymicrobial anaerobic flora, Acinetobacter Cloacae, *S. agalactiae*, *S. maltophilia*, *S. milleri* with *S. lugdunensis*, *S. viridans* with *Prevotella* and *Parvimonas*, *S. anginosus*, and *S. constellatus*. Four instances of *S. pyogenes* were found with *S. Aureus* for the pooled data. Additionally, one was accompanied by *Pseudomonas aeruginosa*. One case was caused by *S. agalactiae*.

**Table 3:** Treatments and outcome.

	Individualized Data		Pooled Data	
	*n*	%	*n*	%
**Treatment**				
Surgical	107		183	
No debridement	4	3.7%	19	10.4%
Single debridement	70	65.4%	108	59.0%
Serial debridements	33	30.8%	56	30.6%
Exenteration/enucleation	12	11.2%	16	8.7%
Other medical therapies	107		183	
Immunoglobulin	4	3.7%	3	1.6%
HBOT	5	4.6%	6	3.3%
NPWT	5	4.6%	5	2.7%
**Outcome**				
Vision	83		154	
Not affected	56	67.5%	104	67.5%
Affected	8	9.6%	22	14.3%
Blindness	19	22.9%	28	18.2%
Septic Shock	38	35.5%	49	26.8%
Death	4	3.8%	9	4.9%

Hyperbaric oxygen therapy (HBOT). Negative pressure wound therapy (NPWT). Vision loss is classified as follows: not affected when no vision loss was reported, affected when vision loss was reported but higher than Hand Movement, and blindness for Hand Movement or less as well as enucleation or exenteration.

**Table 4:** Comparative analysis of risk factors for serial debridement, blindness, septic shock, and death.

		Serial Debridement					Blindness					Septic Shock					Death			
	0 or 1 Debridement		>1 Debridement	*p* Value		Severe Loss		No Severe Loss	*p* Value		No		Yes	*p* Value		No		Yes	*p* Value	
Age	53.3 (21.9)		45.3 (22.6)	**0.083**		57.0 (17.8)		53.1 (18.8)	0.43		46.7 (22.8)		58.4 (19.4)	**0.009**		50.2 (22.4)		71.8 (7.0)	**0.058**	
Sex (Female)	31 (42%)		15 (45%)	0.73		10 (53%)		24 (38%)	0.24		26 (38%)		20 (53%)	**0.14**		42 (41%)		3 (75%)	0.20	
Immunosuppression	36 (49%)		16 (48%)	0.99		12 (63%)		28 (44%)	0.14		31 (45%)		21 (55%)	0.31		48 (47%)		4 (100%)	**0.05**	
DM	15 (20%)		4 (12%)	0.31		4 (21%)		8 (12%)	0.35		12 (17%)		7 (18%)	0.89		15 (15%)		3 (75%)	**0.015**	
Extraocular affection	24 (36%)		17 (52%)	0.13		6 (40%)		26 (42%)	0.89		25 (38%)		16 (46%)	0.48		37 (39%)		4 (100%)	**0.027**	
TOTALS	74		33			19		64			69		38			103		4		

Values represent means and SD or N and percentages. *p* values obtained with *t*-test analysis, chi squared or Fisher exact test. Significant *p* values are in bold.

**Table 5:** Comparison with previous reviews.

	Lazzeri et al. [3]	Amrith et al. [2]	This Work
Year	2009	2013	2025
*N*	103	94	183
Years reviewed	1950 to 2008	1993 to 2012	2013 to 2024
Age (median and range)	50.2 (17 m–93 y)	46.3 (0.1–83 y)	54.2 (8 m–85 y)
Group A beta-hemolytic Streptococcus	68%	51.1%	79.8%
Facial involvement	-	42.6%	45.3%
Debridement	-	85.1%	89.6%
Blindness	-	13.8%	18.2%
Septic shock	-	30.9%	27.2%
Death	14.42%	8.5%	4.9%

## Data Availability

The database generated by this review is not publicly available, but it can be shared upon reasonable request to the corresponding author.
